# Failure to Launch: Predictors of Unfavourable Physical Activity and Sedentary Behaviour Trajectories from Childhood to Adolescence: The Gateshead Millennium Study

**DOI:** 10.3390/ijerph182413283

**Published:** 2021-12-16

**Authors:** Abdulaziz Farooq, Laura Basterfield, Ashley J. Adamson, Mark S. Pearce, Adrienne R. Hughes, Xanne Janssen, Mathew G. Wilson, John J. Reilly

**Affiliations:** 1 Physical Activity for Health Group, School of Psychological Sciences & Health, University of Strathclyde, Glasgow G1 1QE, UK; adrienne.hughes@strath.ac.uk (A.R.H.); xanne.janssen@strath.ac.uk (X.J.); john.j.reilly@strath.ac.uk (J.J.R.); 2 Aspetar, Orthopaedic and Sports Medicine Hospital, FIFA Medical Centre of Excellence, Doha P.O. Box 29222, Qatar; 3 Population Health Sciences Institute, Newcastle University, Newcastle upon Tyne NE2 4AX, UK; laura.basterfield@newcastle.ac.uk (L.B.); ashley.adamson@newcastle.ac.uk (A.J.A.); mark.pearce@newcastle.ac.uk (M.S.P.); 4 Human Nutrition Research Centre, Newcastle University, Newcastle upon Tyne NE4 5PL, UK; 5 Institute for Sport Exercise and Health (ISEH), University College London, London W1T 7HA, UK; Mathew.Wilson@hcahealthcare.co.uk

**Keywords:** children, adolescent, moderate–vigorous intensity physical activity, sedentary behaviour, group-based trajectories, risk factors

## Abstract

In a previous study based on this cohort, only 15% of the participants belonged to a favourable physical activity/sedentary behaviour trajectory group (characterised by relatively high moderate–vigorous intensity physical activity and relatively low sedentary behaviour across childhood and adolescence). Since this favourable trajectory is protective against obesity, we aimed to identify factors associated with membership of this group. In this longitudinal study, 671 participants were assessed at ages 7, 9, 12 and 15 years. Participants’ demographics, socio-economic status (SES) and physical activity environment such as, sports club participation and commuting school were assessed at ages 7, 9 and 12 and analysed with favourable trajectory membership as an outcome using multinomial logistic regression. Sex (male) and SES (higher) were the non-modifiable factors associated with favourable trajectory group. Of the modifiable factors, commuting to school at age 7, a safe environment to play at age 7 and sports club participation at age 12 were all associated with more than 2.0 times increased probability of being in the most favourable trajectory. Future interventions to promote a favourable trajectory could focus on girls and participants with low SES. Promoting active commuting, safe local spaces to play and sports participation should also help lead to a favourable trajectory for physical activity and sedentary behaviour across childhood and adolescence.

## 1. Introduction

During childhood and adolescence, physical activity, particularly moderate-to-vigorous intensity physical activity (MVPA), plays an important role in improving bone health [1], body composition [2,3] and several markers of metabolic health [4]. The World Health Organisation’s (WHO’s) physical activity recommendations for children and adolescents suggest achieving an average of sixty minutes or more MVPA per day, and to limit sedentary behaviour [5]. However, systematic reviews and meta-analyses of longitudinal studies show that MVPA significantly declines from around the age of school entry in both boys and girls [6], while sedentary behaviour increases across childhood and adolescence [7]. Low levels of physical activity might persist into adulthood, and it is understood that sedentary behaviour tracks strongly with age [8,9].

If physical activity and sedentary behaviour are considered within a socio-ecological model, it seems likely that children are exposed to unsupportive environments and so, typically, develop habits that are responsible for an unfavourable physical activity and sedentary behaviour trajectory (i.e., decrease in average time spent in MVPA and increase in average sedentary time with age). Evidence suggests that supportive environments, including school and community programmes, have the potential to increase time spent in children’s free play, structured and unstructured physical activity, and active transport-related behaviours such as walking or cycling to and from school [10,11]. For example, among children, pedometer-assessed physical activity was higher with sports club participation than non-participation [12]. Extensive numbers of studies have focused on individual-level correlates (cross-sectional studies) or determinants (longitudinal studies) of time spent in MVPA and time spent sedentary on obesity outcomes [13,14,15,16,17,18]. In addition to potentially modifiable correlates or determinants of these behaviours and outcomes, some fixed factors have been identified as important correlates or determinants such as sex, socio-economic status (SES) and age. For example, in one study, children with high SES had better cardiorespiratory health and physical activity as compared to children with low SES [19], though in general SES is not associated with objectively measured habitual MVPA in children in the UK [20].

**Figure 1:**
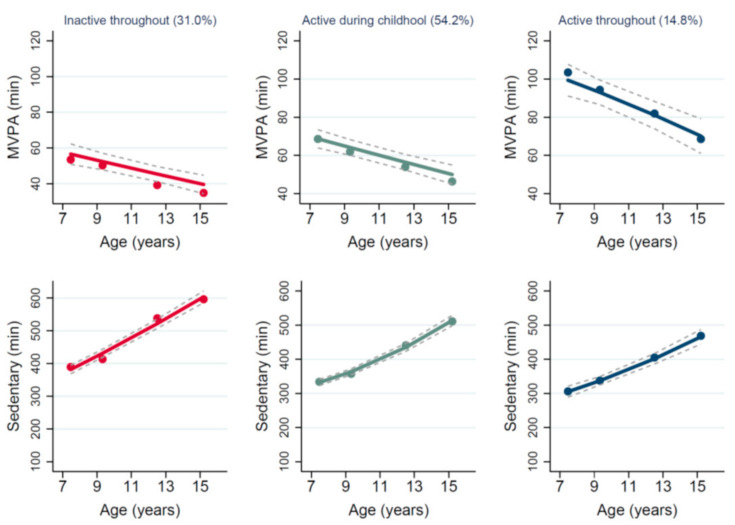
Multiple trajectories of average time spent in moderate–vigorous intensity physical activity (MVPA) and sedentary behaviour (minutes per day) [25].

More recently, the concept of child–adolescent trajectories of MVPA and sedentary time has emerged. These are distinct and recognisable patterns of change with age in the habitual time spent in MVPA [1,6,21,22,23], or habitual time spent sedentary [6], or in trajectories of change in MVPA and sedentary time combined [24,25,26] instead of just simple descriptions of average changes in MVPA and sedentary behaviour. In our previous studies, we have identified such trajectories across childhood and adolescence [25], and have demonstrated that they are associated with different body fatness outcomes towards the end of adolescence [25]. What is less clear is what determines these different trajectories—why do individuals follow a favourable trajectory across childhood and adolescence and who belongs to less favourable inactive trajectories? An understanding that there are distinct latent groups with different trajectories of time spent in MVPA and time spent sedentary provides both quantitative and qualitative information for planning interventions [1,6,21,22,23,26,27]. Recently, in English children, it was found that only a small proportion of children (~15%) belonged to a favourable trajectory group, characterised by maintaining high child and adolescent MVPA (above 60 min/day) and a steady but modest increase in time spent sedentary through adolescence (Figure 1). Being in this favourable trajectory group was associated with much lower adiposity in late adolescence than the other two distinct trajectory groups observed [25]. Since favourable trajectories of MVPA and sedentary time across childhood and adolescence are associated with favourable health outcomes, this study aimed to determine, in English children, whether factors assessed during childhood such as sports club participation, commuting to school, supportive environment and screen time are associated with being in a favourable (MVPA and sedentary time) trajectory across childhood and adolescence, and whether non-modifiable factors such as sex/gender and SES are associated with trajectory group membership [25].

## 2. Materials and Methods

### 2.1. Study Design and Settings

The Gateshead Millennium Study (GMS) was conducted in Gateshead, North East England. More details have been described elsewhere [6,28,29,30]; in brief, this study has a longitudinal cohort study design.

### 2.2. Participants

The sampling procedures and recruitment process of this cohort have been described previously [28,29,31]. In brief, participants were part of a socio-economically representative birth cohort all born in Gateshead (North East England) in 1999/2000, and mostly white (98%).

### 2.3. Measurements

Participants were asked to complete physical activity measures when children were 6 and 8 years old (October 2006 to December 2007) (hereafter referred as age 7). Follow up measurements were at 8–9 years of age (hereafter referred as age 9), at 11–12 years of age (hereafter referred as age 12) and, finally, when children were 14–15 years of age (hereafter referred as age 15).

### 2.4. Anthropometry and Body Composition

The participants had to remove shoes before taking height, which was measured with a precision of up to 0.1 cm using a portable stand (Seca, Birmingham, UK). Weight was measured with the precision of 0.1 kg in light clothing using Tanita TBF300MA (Chasmors, London, UK).

### 2.5. Physical Activity

Objectively measured physical activity and sedentary behaviour was assessed at all four time points (age 7, 9, 12 and 15 years) using accelerometers (ActiGraph GT1M, FL, USA). The methods of assessment have been previously described in detail [25] but, in summary, participants wore the accelerometer above their right hip for 7 consecutive days. Children were requested to wear them at all times during waking hours and were permitted to remove them only for sleep or during water-based activities such as showering, bathing and swimming. The device was set to summarize activity in 15 s (epochs) sampling intervals. A day’s data was considered acceptable if wear time per day was more than 6 h within 24 h [6]. For the physical activity data to be considered valid, at least 3 days (2 weekdays and 1 weekend) of valid data was required. Only those children who provided at least one measurement of valid physical activity data at ages 7, 9, 12 and 15 years were included in this study. The intensity of physical activity was determined using Evenson’s cut-off to define MVPA (574 counts per 15 s) [32]. Duration in sedentary time was computed when the accelerometer reading was <100 cpm, while >10 min of continuous 0 s was considered as non-wear time [32].

### 2.6. Outcome Variable

The main outcome variable for the present study is the probability of belonging to any of the three distinct groups previously identified using multigroup trajectory analysis (Figure 1) [25]. Participants were previously categorized, based on the four measures of accelerometer-measured habitual time spent in MVPA and accelerometer-measured time spent sedentary across childhood and adolescence, into the following three groups: (1) ‘inactive throughout’ (31.0.%), (2) ‘Active during childhood’ (54%), and (3) favourable trajectory, i.e., ‘Active throughout’ (15%). In the ‘inactive throughout’ group, average MVPA per day was below the recommended 60 min at age 7 and declined steeply with age. In addition, time spent in sedentary behaviour was always high relative to the other groups and increased with age. The second group (Figure 1) consisted of 54% of the sample, these individuals were active during childhood with MVPA over 60 min/day, but this declined from age 7, combined with increasing time spent in sedentary behaviour. The third group showed the most favourable trajectory but were also the smallest group (15% of the sample), they had relatively high habitual time spent in MVPA (>60 min/day) across childhood and adolescence, combined with steadily increasing time spent sedentary (Figure 1).

### 2.7. Explanatory Variables

The selection of explanatory variables was based on a combination of the literature on factors influencing accelerometer-measured MVPA and time spent sedentary, and previous findings from the same GMS cohort, which reported, from cross-sectional analyses, that measures of MVPA and sedentary behaviour during childhood are strongly correlated with sex, outdoor play, commuting to school, participation in after school sports clubs and availability of safe places for play at age 7 only [29]. In addition to these variables, we also considered school sports clubs’ participation, which was a predictor for overall physical activity in a longitudinal analysis in the same cohort [33,34]. Since socio-economic status can potentially influence MVPA levels [35] and is positively associated with cardiorespiratory fitness among children [19], it was considered as one of the non-modifiable risk factors.

### 2.8. Non-Modifiable Factors

Demographic data such as participants’ date of birth, sex and SES were collected at birth [29,30]. Sex was based on biological gender recorded at birth. In this study, SES was assessed using the Townsend score. The Townsend score is a validated, area-based measure of socio-economic deprivation. It is computed based on percentage of unemployment, overcrowding and car ownership in the area of residence. It is ranked between 1–5, each corresponding to the 20th, 40th, 60th, 80th and 100th percentile of the general population as a reference [28].

### 2.9. Physical Activity Environment

Parents completed a questionnaire on their child’s physical activity environment at age 7. These questions were based on a five-point Likert scale (1–5; 1 Never, 2 Usually not, 3 Sometimes, 4 Usually, 5 Always) enquiring about how often the child and/or parent engages in different activities (for example: ‘My child walks/cycles from home to school’). For the purpose of analysis, positive responses ‘sometimes’, ‘usually’ and ‘always’ were combined and ‘never’ and ‘usually not’ were combined to create a dichotomous variable. Questions to assess availability of parks/safe outdoor play areas, and whether their child can play unsupervised indoors or outdoors were recorded as true or false. A short version of this questionnaire was administered again when children were 12 years of age to assess school commuting and neighbourhood safety [29,30].

### 2.10. Sports Club Participation

Child participation in sports clubs was enquired directly with children at age 9 and age 12. Children were asked if they participated in school sports clubs or outside school sports clubs. A child was considered to participate in any sports club if he/she participated in either school or outside school sports clubs or both [33].

### 2.11. Statistical Methods

All data were coded and analysed using the statistical software STATA v14.0 (TX: StataCorp LP). The methods used to identify and describe the three distinct trajectory groups have been described previously [25]. In brief, STATA plugin [36] for group-based multiple trajectory analysis was used to identify patterns of trajectories of two dependent continuous variables: time spent in MVPA min/day and time spent in sedentary behaviour min/day jointly [37]. Three groups model provided the smallest Bayesian Information Criteria (BIC) value and, hence, was considered as best fit [38].

To determine the association of categorical variable trajectory group with other categorical explanatory variables at ages 7, 9 and 12, a Chi-square test for independence was used. In order to compare the means of continuous variables Townsend score or parental education with three identified groups, One Way Analysis of Variance (ANOVA) was used. Post hoc comparisons were performed after Bonferroni correction.

In order to identify factors associated with group membership, the identified distinct ordered groups were used as a dependent variable in multinomial logistic regression analysis using the ‘inactive throughout’ group as a reference. Only the significant factors observed from previous univariable analysis were considered in the stepwise backward multinomial logistic regression. The relative risk ratio and 95% CI were reported that provided the risk of belonging to a specific group compared to the ‘inactive throughout’ group (reference group). A *p*-value < 0.1 was used to identify potential factors in univariable analysis, and *p*-value < 0.05 was considered a cut-off for statistical significance in multivariable analysis.

## 3. Results

### 3.1. Characteristics of the Participants

There was an almost equal proportion of boys (49.6%) and girls (50.4%) in the included sample. From the 671 participants, the number of individuals who provided accelerometer data at least once, twice or three times for all four waves were 141, 150, 163 and 217, respectively. Table 1 shows the distribution of SES in the sample. Around 38.8% of the participants were ranked in the 0–40th percentile on the socio-economic status Townsend score, whereas 18.0% were above the 80th percentile on the Townsend score. The average number of years of education for the parents was 14.3 ± 1.9 years.

### 3.2. Univariable Association of Explanatory Variables with Trajectory Group Outcomes

A higher proportion of the ‘active throughout’ group were boys (77.4%) compared to (34.6%) in the ‘inactive throughout’ group (*p* < 0.001). Children in the ‘active during childhood’ group had higher SES (Townsend score) compared to the ‘inactive throughout’ group (*p* = 0.060). Participants whose parents reported their child is interested in sports (*p* = 0.002) and enjoyed active play (*p* = 0.024) at age 8 were more likely to belong to the favourable ‘active throughout’ trajectory group (Table 2).

Participants who commuted from home to school and those whose parents considered that the child has sufficient safe places for play at age 8 and age 12 were more likely to belong to the favourable ‘active throughout’ trajectory group (*p* = 0.027 and *p* = 0.041, respectively). Those children who participated in a sports club (at school or outside school) at age 9 and age 12 were less likely to belong to the ‘inactive throughout group’ (*p* = 0.068 and *p* = 0.043, respectively) (Table 2).

### 3.3. Multivariable Association of Explanatory Factors with Trajectory Group Membership

All significant factors at ages 7, 9 and 12 that were associated with multi-trajectory group membership in Table 2 were entered in a multinomial logistic regression using ‘inactive throughout’ as a reference category. Following a stepwise backward elimination, multinomial logistic regression only significant factors were retained (Table 3). Table 3 shows that boys were 2.3-fold more likely to be a member of the ‘active during childhood’ group (RR 2.3, 95% CI (1.3–4.0)) and 8.2-fold more likely to be in the favourable ‘active throughout’ trajectory (RR 8.2, 95% CI (3.7–18.4)) compared to being in the ‘inactive throughout’ group. One unit of higher socio-economic status increased the chances of being in the ‘active during childhood’ and favourable trajectory groups by 35% and 42%, respectively (Table 3). The child commuting to school at age 7 and having a safe environment for play at age 7 resulted in more than a 2.5 times increased chance of belonging to the favourable trajectory group. School sports club participation at age 12 was significantly associated with the favourable trajectory group (RR 2.5, 95% CI (1.2–5.4)).

Dots indicate the observed average MVPA and sedentary behaviour (minutes/day), and solid lines are trajectories showing the estimated mean time spent in MVPA and sedentary behaviour (minutes/day) across age for the three groups identified. The grey dashed lines represent the 95% confidence intervals for each trajectory.

## 4. Discussion

The WHO 2020 guidelines on physical activity for school-age children and adolescents emphasise time spent in MVPA and time spent sedentary. In our previous study, we established three identifiable trajectories of MVPA and sedentary time across childhood and adolescence in England. Membership of the trajectory groups strongly influenced body fatness in late adolescence [25]. Since these distinct trajectories of MVPA and sedentary time are important to body fatness, it is useful to identify the factors influencing membership of one trajectory group rather than another.

### 4.1. Main Findings

In the present study, the factors that influenced membership of the trajectory groups in multivariable models were a mixture of modifiable (active commuting to school, sports club membership, safe environment for outdoor play) and non-modifiable (sex, SES) factors. The present study suggests that these childhood factors have a long-lasting influence on time spent in MVPA and sedentary behaviour (and on related health outcomes such as fatness) across childhood and adolescence, and so will be useful in planning future physical activity/sedentary behaviour interventions.

To the best of our knowledge there are few studies that have used a multi-trajectory approach to model two forms of contrasting but related movement behaviours, daily MVPA and sedentary time, across childhood to adolescence and to study lifestyle-related factors that predicted group membership. As noted above, we are unaware of any longitudinal studies that identified modifiable and non-modifiable factors associated with combined trajectories of MVPA and sedentary time across childhood and adolescence. However, a brief consideration of the present study findings in the context of the previous literature is discussed.

### 4.2. Gender

While the majority of children and adolescents of both sexes in the UK have low MVPA and spend large parts of their day sedentary, and declines in MVPA and increases in time spent sedentary affect both sexes by early–mid childhood [6,7], this is not the same as the more nuanced issue of possible differences between boys and girls in the probability of belonging to particular trajectories of time spent in MVPA and time spent sedentary across childhood and adolescence. Changes in overall physical activity from age 10 to age 16 via group-based trajectory analysis in US youths reported two factors; being male (77.8%) and having a lower BMI at age 10 years [39]. In our study, sex of the child was the most important factor for high MVPA and low sedentary time group membership. In our study, the most active were also most likely to be boys (77.4%), but BMI at age 7 or age 9 were not associated with active trajectory membership. 

### 4.3. Socio-Economic Status

Low socio-economic status in childhood can have a range of effects including differential access to health care, different physical activity environment and differential exposure to stress [40], but many studies that have measured habitual MVPA objectively in UK children have found negligible differences between children of different SES. The present study examined SES influences on differences in MVPA and sedentary behaviour trajectory across childhood and adolescence, a subtle but important difference compared to previous studies. After adjustment, a unit increase in Townsend quintile score was significantly associated with higher probability of being in ‘the active throughout group’. In Australian pre-adolescents, there was high amount of MVPA among children with higher socio-economic status [41]. Similarly, in US children, boys and girls with higher household income were 2.0 and 2.6 times more likely to achieve physical activity guidelines during adulthood, respectively [42]. Children with low socio-economic status generally have low participation in school and community sports activities [43], though not habitual MVPA as measured by accelerometery. While SES has negligible influence on time spent in MVPA at single points in time in UK studies, including our previous analyses of the Gateshead Millennium Study cohort [20], SES might influence trajectories via an influence on sustained participation in organised sport and physical activity.

### 4.4. Sports Club Participation

In a previous report of the same cohort (GMS), sports participation at age 12 was associated with a 5% increase in MVPA and a 4% reduction in sedentary behaviour. By applying a multi-trajectory approach, we were able to see that children who did not participate in school sports clubs at age 9 and age 12 were at a significantly higher risk of being in the ‘inactive throughout’ group in the univariable analysis. The absence of outside school sports clubs as a contributor to trajectories is maybe because the preferred outside school activity among participants was swimming (35.3% at age 9 and 9.1% at age 12), which goes undetected with accelerometers. The multivariable analysis identified school sports club participation as a meaningful contributor to the ‘active throughout’ group membership. This finding is consistent with results from the Australian LOOK longitudinal study, which provided evidence that sport participation is an important vehicle to increase MVPA and reduce sedentary time in children and adolescents aged 8–16 years [12]. Moreover, in the Australian study, sports participation improved cardiorespiratory fitness and reduced body fat development in girls and increased the chance of meeting physical activity recommendations in children over the period 8 to 16 years [12]. Sports club participation at least once a week was associated with 2.5- and 3.5-fold increases in the achievement of physical activity recommendations in 6–10 year olds and 11–17 year olds, respectively [44]. In Irish youths, sports participation positively predicted physical activity for the next five years [45]. In fact, a 28 year follow-up study showed that participants who were active in youth sports had a higher chance of healthy lifestyle behaviour, especially among the women [46]. Unfortunately, recent reports suggests that sports club participation declines during the transition from childhood to adolescence [47].

The summary above concerns associations between sports club participation and MVPA only, not associations with trajectories of MVPA and/or sedentary behaviour. We are only aware of a single study that attempted to examine associations between sports club participation in children and adolescents and MVPA trajectory, and that study did not consider sedentary behaviour trajectory [42]. That study investigated sports participation trajectories in US youths from 6 to 18 years and found three distinct groups, the continuously participating group was 45.9% and dropouts from sports participation occurred mostly between 9 to 13 years [42].

### 4.5. Active Commute to School

In our study, the prevalence of active commuting declined from age 9 to 12, and prevalence was higher in boys and children with lower socio-economic status, confirming recent findings [48]. It is evident that active commuting is associated with lower BMI [49,50]. Studies from many different regions around the world [51,52,53] suggest that active commuting to school can increase MVPA among children and adolescents. There is a secular decline in the percentage of children who actively commute to school [54]. Via active commuting, an additional 24 to 45 min of MVPA can be accumulated per day [55,56] and, hence, commuting to school can be a main source of activity among children when they do not participate in a sports club. These previous studies did not consider the influences of the commuting mode on the trajectories of MVPA and sedentary behaviour, the focus of the present study. In our study, children who commuted to school at age 7 were 2 times more likely to belong to the active throughout trajectory. Recently, schools that implemented policies and interventions to support active commuting have found positive changes in body composition in the early school years [57,58]. However, the commute distance should probably be more than 0.8 km to see a meaningful change in daily activity [57].

### 4.6. Safe Environment

Commuting to school and outdoor time is often associated with a clustering of other environmental factors such as a safe neighbourhood and safe traffic amenities [59,60]. It has previously been reported that having access to a green park and high perceived traffic safety increased daily MVPA by 6.8 and 2.5 min per day, respectively, among 6–10-year-old children. In a study involving Mexican children, the perceived pedestrian safety among older boys aged 15–18 years was not associated with MVPA or sports participation, however, pedestrian safety was not associated with MVPA among girls [61,62]. We are not aware of any previous studies of perceived neighbourhood safety influences on the trajectories of MVPA, trajectories of sedentary behaviour, or trajectories of MVPA and sedentary behaviour combined. In our study, having a perceived safe physical activity environment at age 7 was associated with the most favourable active throughout trajectory group. In the regression analysis performed in our study, perceived neighbourhood safety when children were 12 years was no longer a significant contributor to the active trajectory group membership. This could imply that perceptions of safety are most important at a younger age, possibly because that is when MVPA and sedentary behaviour habits are being formed.

### 4.7. Strengths and Limitations

The main strength of this study was the longitudinal study design, recruiting participants in four waves from age 7 to age 15 years. Another feature that makes this study stronger was the fact that physical activity was monitored objectively at each wave using accelerometers, and many known modifiable and non-modifiable risk factors were assessed at key intervals during the child’s growth. The uniqueness that makes it standout from other studies is the innovative way of analysing the data using multi-trajectories for MVPA and sedentary behaviour. As noted above, no previous study has examined the determinants of combined MVPA and sedentary behaviour trajectories across childhood and adolescence. We believe that by presenting the size of a favourable trajectory group and by identifying significant factors associated with this trajectory, we are able to communicate more effectively with the policy makers and general audience. Our three distinct trajectory groups, previously identified as influencing body fatness in late adolescence [25], were novel and, therefore, a strength, and we had adequate sample size per group to draw meaningful information from this study, which was another strength.

However, this study does have a few limitations to consider. The first limitation of this study is the loss of follow-up among children at age 15 y: 21.1% of participants had missing or provided invalid accelerometer data. However, this was less likely to result in bias because physical activity measures and BMI-z scores at baseline among participants that provided data were comparable to those who had missing accelerometer data. Another limitation to this study could be that, since pubertal maturation status was not assessed, it was not possible to include biological influences along with environmental factors associated with active lifestyles through adolescence. Even though there were four waves of data collection, the information regarding participation in sports clubs, commuting to school and perception of neighbourhood safety was not assessed at every time point. Screen time behaviour is changing very swiftly with emerging technology, ease of access and academic demands. These behaviours must be frequently monitored, possibly even on an annual basis, given the developments in new technology and children’s access to screens, in order to provide meaningful analysis. Despite these limitations, data regarding sports participation and commuting to school were assessed across the relevant age groups, which covered much of childhood and adolescence (age 7, 9 and age 12).

## 5. Conclusions

The present study found a number of fixed and modifiable variables that determined the trajectories of time spent in MVPA and time spent in sedentary behaviour across childhood and adolescence. The fixed variables, gender and socio-economic status, might be useful in identifying children at high risk of being in an unfavourable trajectory from early life. The modifiable variables such as active commuting to school, the provision of safe and adequate space for physical activity participation and sports club membership at school or after school could potentially shift children into more favourable trajectories, with long-lasting impacts across childhood, adolescence and adulthood.

## Figures and Tables

**Table 1:** Characteristics of the sample.

	N	%
Sex		
Boys	333	49.6%
Girls	338	50.4%
Townsend Score Quintile		
0–19	118	17.7%
20–39	140	21.1%
40–59	153	23.0%
60–79	134	20.2%
80–100	120	18.0%

**Table 2:** Modifiable and non-modifiable factors at age 7, 9 and 12 years in association with identified distinct trajectory groups for MVPA and sedentary behaviour combined (Univariable analysis).

Explanatory Variables	Inactive Throughout (N = 185)	Active during Childhood (N = 393)	Favourable Group (Active Throughout) N = 93	*p*-Value
Sex				
Males	34.6%	50.1%	77.4%	<0.001
Females	65.4%	49.9%	22.6%	
Townsend Score	2.8 ± 1.3	3.1 ± 1.4	2.9 ± 1.3	0.060
Physical Activity Environment at Age 7 ^‡^				
Given the choice, my child would rather play an active game than watch TV/play a computer game	105(56.8)	256(65.1)	65(69.9)	0.057
My child is interested in sport	125(67.6)	298(75.8)	81(87.1)	0.002
My child likes to try new games and sports	137(74.1)	299(76.1)	80(86.0)	0.069
My child prefers reading to active play	50(27.0)	105(26.7)	15(16.1)	0.089
My child enjoys taking part in after-school games clubs	126(68.1)	276(70.2)	71(76.3)	0.359
My child enjoys active play	162(87.6)	363(92.4)	90(96.8)	0.024
I play with my child if he/she asks me to join in	148(80.0)	296(75.3)	68(73.1)	0.345
I take exercise at the same time as my child	56(30.3)	137(34.9)	24(25.8)	0.190
My child helps with housework/in the garden	91(49.2)	187(47.6)	32(34.4)	0.046
I try to be more active so my child is more active	91(49.2)	192(48.9)	40(43.0)	0.565
I think it is important for my child to be active	180(97.3)	380(96.7)	87(93.5)	0.257
I think my child is active at school	170(91.9)	370(94.1)	89(95.7)	0.407
I would like my child to be more active	86(46.5)	164(41.7)	30(32.3)	0.076
I think my child gets enough activity at school and doesn’t need to be active at home	48(25.9)	108(27.5)	16(17.2)	0.124
My child walks/cycles from home to school	110(59.5)	272(69.2)	68(73.1)	0.027
I think there are enough places for my child to play safely	33(22.0)	55(17.9)	23(28.0)	0.187
I would like to see more play areas near my home	110(73.3)	220(71.4)	61(74.4)	0.920
I consider it to be safe for my child to play outside	36(24.0)	93(30.6)	35(43.2)	0.044
I have a yard/garden my child can play in	147(97.4)	299(97.1)	81(98.8)	0.855
My child can play unsupervised indoors	116(76.8)	232(75.3)	59(72.0)	0.864
My child can play unsupervised outdoors	45(29.8)	124(40.4)	37(45.1)	0.176
During the last week has your child done any sport and exercise activities	63(71.6)	130(62.8)	38(73.1)	0.191
Participates in Sports Club (Age 9) ^†^	**Inactive Throughout**	**Active during Childhood**	**Active Throughout**	***p*-Value**
At school	45(29.6)	121(36.8)	35(43.8)	0.087
Outside school	85(56.3)	192(58.5)	54(66.7)	0.293
Physical Activity Environment (12 y) ^†^				
My child walks/cycles from home to school	44(32.8)	120(43.5)	36(52.9)	0.016
I think there are enough places for my child to play safely	52(28.3)	83(21.4)	30(32.3)	0.041
I would like to see more play areas near my home	79(42.9)	182(46.8)	43(46.2)	0.683
I consider it to be safe for my child to play outside	74(40.2)	146(37.4)	44(47.3)	0.212
Participates in Sports Club (Y12) †				
At school	42(22.7)	125(31.8)	37(39.8)	0.009
Outside school	61(33.0)	141(35.9)	42(45.2)	0.131

^‡^ The frequency and (%) percentages refer to participants who responded positively (sometimes, usually or always); ^†^ The frequency and (%) percentages refer to participants who responded affirmative (Yes).

**Table 3:** Multinomial logistic regression analysis showing the relative risk ratios (RR) and 95% confidence intervals for the factors associated with trajectory group membership from 7 to 15 years.

		**Active during Childhood**		**Active Throughout**	
**Explanatory Variables**	Inactive Throughout *	**RR (95% CI)**	***p*** **-Value**	**RR (95% CI)**	***p*** **-Value**
Sex: Boys	1.0	2.31(1.33–4.01)	0.003	8.20(3.65–18.41)	<0.001
Townsend Quintile	1.0	1.35(1.09–1.67)	0.005	1.42(1.07–1.88)	0.016
Age 7					
Child commutes to school	1.0	2.26(1.31–3.91)	0.003	2.56(1.18–5.56)	0.018
Safe environment for play	1.0	1.50(0.83–2.70)	0.179	2.65(1.22–5.76)	0.014
Age 9					
School Sports Club Participation	1.0	1.59(0.89–2.83)	0.114	2.14(0.99–4.64)	0.054
Age 12					
Child commutes to School	1.0	1.60(0.89–2.85)	0.114	2.24(1.03–4.88)	0.043
School Sports Club Participation	1.0	1.48(0.86–2.57)	0.160	2.51(1.18–5.35)	0.017

* Reference category.

## Data Availability

The datasets used and/or analysed during the current study are available from the corresponding author on request.
